# CD73-positive extracellular vesicles promote glioblastoma immunosuppression by inhibiting T-cell clonal expansion

**DOI:** 10.1038/s41419-021-04359-3

**Published:** 2021-11-09

**Authors:** Ming Wang, Jiaoying Jia, Yan Cui, Yong Peng, Yugang Jiang

**Affiliations:** Department of Neurosurgery, The Second Xiangya Hospital of Central South University, Changsha, 410011 Hunan China

**Keywords:** CNS cancer, Tumour immunology

## Abstract

Extracellular vesicles are involved in the occurrence, progression and metastasis of glioblastoma (GBM). GBM can secrete a variety of tumour-derived extracellular vesicles (TDEVs) with high immunosuppressive activity that remotely suppress the systemic immune system, and therapy targeting TDEVs has potential efficacy. In this study, we detected a higher concentration of CD73+ TDEVs enriched in exosomes in central and peripheral body fluids of GBM patients than in those of patients with other brain tumours (low-grade glioma or brain metastases from melanoma or non-small-cell lung cancer). High CD73 expression was detected on the surface of T cells, and this CD73 was derived from TDEVs secreted by GBM cells. In vitro, we observed that CD73+ TDEVs released by GBM cell lines could be taken up by T cells. Moreover, excess adenosine was produced by AMP degradation around T cells and by adenosine receptor 2A (A_2A_R)-dependent inhibition of aerobic glycolysis and energy-related metabolic substrate production, thereby inhibiting the cell cycle entry and clonal proliferation of T cells. In vivo, defects in exosomal synthesis and CD73 expression significantly inhibited tumour growth in GBM tumour-bearing mice and restored the clonal proliferation of T cells in the central and peripheral regions. These data indicate that CD73+ TDEVs can be used as a potential target for GBM immunotherapy.

## Background

Immune checkpoint protein inhibitor therapy (ICT), such as antibodies against PD-L1 or PD-1, has shown effectiveness against a variety of cancers, including skin cutaneous melanoma (SKCM), non-small cell lung cancer (NSCLC) and breast cancer, with many patients who previously failed a variety of other treatment strategies achieving sustained remission. However, in some malignant tumours characterised by systemic immunosuppression, such as glioblastoma (GBM), the conventional chemotherapeutic drug temozolomide (TMZ) aggravates the systemic immunosuppression observed in GBM, which often results in ICT failure in the treatment of GBM [1]. A variety of programmes aimed at improving the immunosuppressive properties of GBM, such as inhibiting the expression of CD73 [2–4] and CD39 [5], have been developed.

Extracellular vesicles (EVs) are widely present in the extracellular microenvironment and perform material transfer and information exchange between cells. Tumour-derived extracellular vesicles (TDEVs) promote the survival and proliferation of cancer cells through paracrine signalling. In addition, the remote regulatory effects of TDEVs, such as activation and promotion of epithelial–mesenchymal transition, niche formation before metastasis, and remote immune regulation [6], deserve attention. By harbouring immunomodulatory molecules, TDEVs can change the immune microenvironment, which may impact the effects of conventional chemotherapy and ICT. In our previous studies, TDEVs from GBM patients were found to have the unique protein ligand LGALS9, which inhibits antigen recognition, processing and presentation by dendritic cells (DCs), leading to failure of the cytotoxic T cell-mediated antitumour immune response [7]. CD73 is encoded by the enzyme ecto-5′-nucleotidase (*NT5E*) and is primary induced by hypoxia [8]. CD73 supports cancer progression and protects tumours from immune surveillance and chemical resistance by catalysing the synthesis of adenosine [3, 9]. CD73 is highly expressed in GBM and has been confirmed to be associated with multiple elements of pathogenesis, including growth, angiogenesis, and invasiveness [4]. CD68+ macrophages with high CD73 expression have been detected in GBM, and these cells show strong immunosuppressive activity and can be used as a target in combined ICT for GBM therapy [10].

In our preliminary experiments, we found high levels of CD73+ TDEVs in the cerebrospinal fluid (CSF) of GBM patients [7]. However, whether CD73+ TDEVs can promote GBM progression by inducing local and/or systemic immunosuppression is unclear. Therefore, in this study, we focused on investigating the role of CD73+ TDEVs in the CSF of GBM patients and the impact on the GBM immune microenvironment, as well as the effect of inhibiting CD73+ TDEVs on the pathological progression of GBM.

## Materials and methods

### Subjects and cohorts

All procedures performed in this study involving human participants complied with the ethical standards of the institution and/or the National Research Council and with the 1964 Declaration of Helsinki and its subsequent amendments or similar ethical standards. Within 24 months (from February 2019), patient tissues, CSF and blood were prospectively collected. All brain tumour patients provided informed consent for participation in the study, and the experimental protocol was carried out under the approval of the ethical review committee of The Second Xiangya Hospital of Central South University. The cases, which included primary World Health Organisation grade IV GBM (*n* = 17), grade II–III glioma (diffuse astrocytoma (*n* = 9) and anaplastic astrocytoma (*n* = 4)) and metastatic brain tumours (SKCM (*n* = 6) and NSCLC (*n* = 4)), were confirmed histopathologically. Table S1 provides detailed information for all subjects.

### Cell lines

The mouse glioma cell line GL-261 and the human malignant brain astroglioma cell lines U-251 MG, U-87 MG and U-118 MG were obtained from the Kunming Cell Bank of the Type Culture Collection Committee of the Chinese Academy of Sciences (Sigma-Aldrich, MO, USA). Primary HAs were obtained according to a previous method [7]. GL-261 and U-118 MG cells were cultured in exosome-free Dulbecco’s modified Eagle’s medium (DMEM; HyClone, UT, USA) containing 10% exosome-depleted foetal calf serum (HyClone, UT, USA) and 1% penicillin-streptomycin (Sigma-Aldrich, MO, USA) at 37 °C and 5% CO_2_. U-251 MG and U-87 MG cells were cultured in exosome-free Minimum Essential Medium (MEM) (HyClone, UT, USA) containing 1% non-essential amino acids (Sigma-Aldrich, MO, USA), 10% exosome-depleted foetal bovine serum (HyClone, UT, USA) and 1% penicillin-streptomycin (Sigma-Aldrich, MO, USA) at 37 °C and 5% CO_2_. For T-cell sorting and purification, whole-blood samples were obtained from the blood centre of ChangSha (Hunan, China) under a protocol approved by the ethics review committee. Briefly, blood samples were placed in vacutainer tubes (Becton Dickinson, UK) containing EDTA (Sigma-Aldrich, MO, USA), and peripheral blood mononuclear cells (PBMCs) were isolated using Histopaque-1077 (Sigma, Dorset, UK). CD3+ T cells were purified using immunomagnetic beads (Miltenyi Biotec, Surrey, UK), and PBMCs in suspension were sorted using a FACSCanto flow cytometer according to the manufacturer’s instructions.

### Tumour-bearing mice

C57BL/6J mice (Laboratory Animal Centre of Fudan University Shanghai, China) were bred under pathogen-free conditions, and 8- to 10-week-old mice were used for tumour experiments. Animal experiments were performed under the approval of the Animal Care and Use Committee of The Second Xiangya Hospital of Central South University. Mice that died of non-tumour-related reasons were eliminated, and mice were euthanized when the tumour grew to ≥1 cm^3^ in diameter or at 35 days after tumour cell injection. According to the method, 2.5 × 10^5^ WT GL-261, CD37^−/−^GL-261, Rab27a^−/−^GL-261 or nSMase2^−/−^GL-261 cells were implanted in the mouse brain. On the 14th day, the mice were euthanized, and blood and CSF were collected to evaluate CD73+ TDEVs and perform adenosine ELISAs (Anogen, Ontario, Canada). On day 28, T-cell analysis was performed to evaluate the tumour tissue, CSF and blood of tumour-bearing mice.

### Flow cytometry and western blotting

Pathological tissue from a surgically resected brain tumour patient was cut into small pieces <1 mm^3^, added to 10 mL of DMEM (HyClone, Logan, UT, USA) containing 2 mg/mL collagenase A (Sigma-Aldrich, MO, USA) and 1X DNase I Sigma-Aldrich, MO, USA), and homogenised at 37 °C for 30 min. Foetal calf serum (HyClone, UT, USA) was added to terminate the digestion, and 70-, 40- and 20-μm nylon mesh filters (Millipore Corp., MA, USA) were used to filter the homogenate. The cells were then centrifuged at 4800 × *g* for 15 min at 4 °C, and the cell pellet was resuspending in 1% BSA. For CSF and blood processing, cells were treated with red blood cell lysis buffer (Beyotime, Shanghai, China) and centrifuged at 3600 × *g* and 4 °C for 15 min to remove cell debris, and the cell pellet was resuspended in 1% BSA. According to the recommended dilution ratio, a variety of fluorophore-conjugated anti-human antibodies were incubated with the cell suspension for 2 h in the dark, and then myeloid and lymphoid cells were detected using a BD Biosciences flow cytometer (Franklin Lakes, NJ, USA). Western blotting was used to detect the expression of CD73, cell- and exosome-related markers, and key metabolic enzymes in surgically resected tissues. In short, collected tissue digestion supernatants and cells were treated with a cell lysis buffer (Beyotime, Shanghai, China) on ice for 2 h, added to 1:1 loading buffer and boiled for 15 min to expose target antigens. The samples were loaded on a 12% separating gel and 6% concentrated gel, and electrophoresis was performed at 120 V for 120 min. The proteins in the separating gel were transferred to a nitrocellulose (NC) membrane (380 mA, 2 h) (Beyotime, Shanghai, China) via a wet method, and the NC membrane was blocked with 5% BSA for 2 h. After washing, the membrane was incubated with the recommended dilution of a primary antibody at 4 °C overnight. After washing, a corresponding HRP-labelled secondary antibody was incubated at room temperature for 1 h, DAB was used for development, and a chemiluminescence system was used for imaging (Bio-Rad, CA, USA). Table S2 shows the antibody information required for flow cytometry and Western blot experiments.

### Isolation and characterisation of exosomes

TDEVs from the CSF or blood of brain tumour patients or tumour-bearing mice were isolated by centrifugation at 720 × *g* and 4800 × *g* for 10 min to remove cells and cell debris, followed by ultrafiltration centrifugation (120,000 × *g*, 120 min) to obtain TDEVs smaller than 100 nm (0.1 μm) (Filter sieve, Sigma). Then, a NanoSight (Malvern Panalytical, Malvern, UK) was used to count EVs and determine their size of; NTA 3.1 software was used to analyse video recordings, and imaging was performed three times for each sample. A transmission electron microscope was used to image EVs, and isolated EVs were suspended in 30 μL of PBS at 4 °C. Eluted exosomes were adsorbed onto a Formvar-carbon-coated electron microscope grid and imaged at 80 kV under a Tecnai G 2 Spirit BioTWIN microscope.

### Analysis of intermediate metabolites

Liquid chromatography/mass spectrometry (LC/MS) was performed on a Thermo Fisher Scientific Triple Quadrupole LC–MS system. After pre-incubation with TDEVs for 6 h, cells were stimulated with αCD3 + αCD28 (20 ng/ml; Sigma-Aldrich, MO, USA) for 3 days. Then, the cells were collected, and the cell pellet was washed with ice-cold PBS. One millilitre of ice-cold 80% methanol containing 10 ng/ml internal standard valine-d8 was used to extract polar metabolites. After centrifugation at 24,000 × *g* for 10 min, the sample was dried in a benchtop vacuum centrifuge and dissolved in 100 mL of water; 1 mL of sample was injected into a ZIC-pHILIC 2.1 × 150 mm chromatographic column (EMD Millipore). The buffer was 20 mM ammonium carbonate + 0.1% ammonium hydroxide (Buffer A) and acetonitrile (Buffer B). The chromatographic gradient was performed at a flow rate of 0.2 ml/min. XCalibur QuanBrowser 2.2 (Thermo Fisher Scientific, MA, USA) was used to reference an internal chemical standard library for relative quantification of polar metabolites. The relative abundances of intermediate metabolites were obtained by normalising the analysis results and comparing the number of cells.

### CRISPR/Cas9 knockout cell line and a fluorescently tagged CD73/CD63 protein overexpression plasmid vectors

According to a standard CRISPR/Cas9 knockout protocol, an sgRNA oligonucleotide (GenePharma, Shanghai, China) specific for the target gene was cloned into the pLVX-IRES-mCherry vector (GenePharma, Shanghai, China), and two different sgRNA guides were designed for each gene. One microgram of each plasmid was transfected into U-87 MG, GL-261 or T cells using Lipofectamine 3000 (Thermo, MA, USA). After 48 h, the transfection efficiencies of CD73^−/−^, Rab27a^−/−^ and nSMase2^−/−^ U-87 MG cells and A_2A_R^−/−^ T cells were detected by evaluating the mCherry fluorescence intensity; cell transfection efficiency was detected by Western blotting or flow cytometry to evaluate protein knockout. For overexpression vectors, the CDSs of the CD73 and CD63 genes were provided by Clontech (CA, China) and ligated into the pcDNA3.1-GFP (Thermo, MA, USA) and pcDNA3.1-mCherry (Thermo, MA, USA) overexpression vectors. Lipofectamine 3000 (Thermo, MA, USA) was used to transfect 1 µg of each plasmid into HA, U-87 MG, U-118 MG and U-251 MG cells, and the fluorescence intensity was measured to verify the transfection efficiency. The sgRNA sequences are shown in Table S3.

### PCR detection of NT5E expression

Total RNA was isolated from CD45- cells, CD45+ cells, CD4+ T cells and CD8+ T cells from brain tumour patients sorted by flow cytometry with QIAzol reagent (QIAGEN, Hilden, Germany). 5 × FastKing-RT SuperMix (Thermo, MA, USA) was used to reverse transcribe the isolated RNA into cDNA, SYBR green (Thermo Fisher Scientific, Waltham, MA, USA) was used on a real-time quantitative PCR system (Applied Biosystems, CA, USA) for NT5E mRNA quantification; GAPDH was used as the internal reference. The following primers were used: NT5E forward primer: 5′-GCCTGGGAGCTTACGATTTTG-3′, NT5E reverse primer: 5′-TAGTGCCCTGGTACTGGTCG-3′, GAPDH forward primer: 5′-GGGTGATGCAGGTGCTACTT-3′, and GAPDH reverse primer: 5′-GGCAGGTTTCTCAAGACGGA-3′.

### Indirect ELISA method to determine the TDEV concentration

An ELISA method was used to detect the concentration of CD73+ TDEVs in the CSF or blood of patients and mice. First, an ELISA plate was coated with a monoclonal anti-mouse or anti-human CD73 antibody (Invitrogen, CA, USA) (0.25 μg per well) for an adsorption immunoassay. ELISA blocking buffer (Anogen, Ontario, Canada) was used to block non-specific binding sites at room temperature. Then, 50 µl of centrifuged (3600 × *g* at 4 °C for 15 min) CSF or blood supernatant samples were added to each well and incubated overnight at 4 °C. The monoclonal anti-mouse or anti-human CD73 antibody was added to the ELISA plate wells for secondary immunocoupling and incubated for 2 h at room temperature. After washing with PBS, an HRP-labelled secondary antibody (Beyotime, Shanghai, China) was added and reacted with a TBM chromogenic solution (Beyotime, Shanghai, China). The absorbance intensity at 450 nm was measured. A standard curve was prepared using a recombinant CD73 protein (Invitrogen, CA, USA), and a recombinant CD68 protein (Invitrogen, CA, USA) was used as a negative control.

### Statistical analysis

Data were analysed using one-way analysis of variance or a paired Student’s *t* test. Statistical analyses and graphical representation of the data were performed using GraphPad Prism 6.0 (GraphPad Inc.) and SPSS version 19.0 (IBM Corp.). *P* < 0.05 was considered to indicate a statistically significant difference.

## Results

### GBM patients secrete high levels of CD73+ TDEVs

**Fig. 1:**
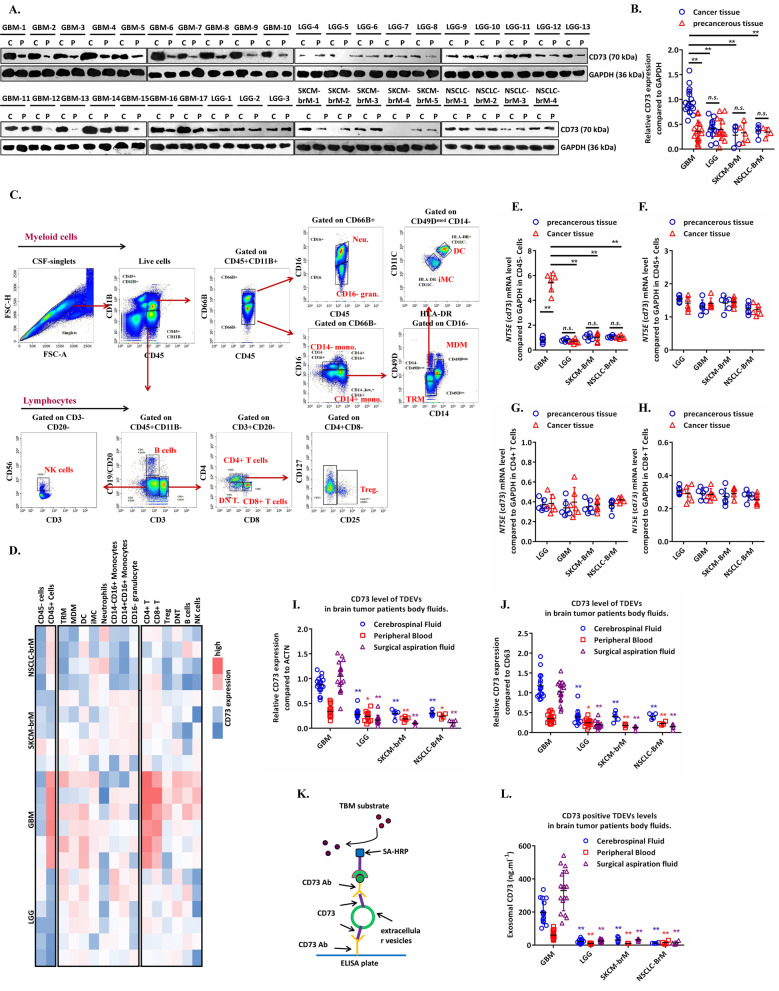
The origin of CD73-positive TDEVs in body fluids of brain tumour patients. **A** The expression level of CD73 in surgically removed cancer tissues and adjacent tissues from patients with brain tumours (*n*_GBM_ = 17, *n*_LGG_ = 13, *n*_SKCM-BrM_ = 5, and *n*_NSCLC-BrM_ = 4) was detected by Western blotting (P paracancerous tissue, C cancer tissue). **B** Graph of relative CD73 protein expression, related to (**A**). **C** Representative patterns of myeloid immune cells [tumour-resident macrophages (TRMs), monocyte-derived macrophages (MDMs), dendritic cells (DCs), immature myeloid cells (iMCs), neutrophils, CD14^−^CD16^+^ monocytes, CD14^+^CD16^+^ monocytes, and CD16^−^ granulocytes] and lymphoid immune cells [CD4+ T cells, CD8+ T cells, regulatory T (Treg) cells, double-negative T cells (DNTs), B cells and nature killer (NK) cells] in tumour tissues from brain cancer patients. **D** Average fluorescence intensity of CD73 in CD45^−^ cells, CD45^+^ cells, myeloid cells and lymphoid immune cells in tumour tissues from patients (*n*_GBM_ = 6, *n*_LGG_ = 6, *n*_SKCM-BrM_ = 5, and *n*_NSCLC-BrM_ = 4). **E**, **F** The relative levels of CD73 (*NT5E*) mRNA in sorted CD45^−^ cells and CD45^+^ cells in tumour and adjacent tissues from brain tumour patients (*n*_GBM_ = 6, *n*_LGG_ = 6, *n*_SKCM-BrM_ = 5, and *n*_NSCLC-BrM_ = 4). **G**, **H** The relative levels of CD73 (*NT5E*) mRNA in sorted CD4^+^ T cells and CD8^+^ T cells in tumour tissues and adjacent tissues from brain tumour patients (*n*_GBM_ = 6, *n*_LGG_ = 6, *n*_SKCM-BrM_ = 5, and *n*_NSCLC-BrM_ = 4). **I**, **J** Western blotting analysis of CD73 expression levels in EVs from body fluids of brain tumour patients; CD63 was used as the exosome internal control, ACTN was used as the Pan-EV control, and the BCA method was used to determine the EV loading volume. Related to Supplementary Fig. 1A. (*n*_GBM_ = 17, *n*_LGG_ = 13, *n*_SKCM-BrM_ = 5, and *n*_NSCLC-BrM_ = 4). **K** Modified ELISA method to detect CD73-positive EVs in body fluids from patients. **L** The concentration of CD73-positive TDEVs in cerebrospinal fluid, peripheral blood and surgical aspiration fluid from brain tumour patients (*n*_GBM_ = 17, *n*_LGG_ = 13, *n*_SKCM-BrM_ = 5, and *n*_NSCLC-BrM_ = 4). Data are shown as the mean ± SD; *t* test, *p < 0.05 and **p < 0.01 compared to the GBM group in (**I**, **J**, **L**).

Compared with breast cancer, SKCM and non-squamous NSCLC [11], GBM presents additional challenges for achieving a therapeutic effect with ICT [1, 12], which seems to be related to the low expression of CD73. For this reason, we collected tumour tissues of SKCM brain metastases (SKCM-brM, 6 cases), NSCLC brain metastases (NSCLC-brM, four cases), grade II–III low-grade glioma (LGG, 13 cases) and GBM (17 cases). The expression of CD73 in GBM tumour tissue was significantly higher than that in SKCM-brM, NSCLC-brM and LGG and was higher than that in adjacent tissues (Fig. 1A, B). Flow cytometry was used to analyse the subpopulations of myeloid and lymphoid immune cells in these tumour tissues. As shown in Fig. 1C, CD73 was mainly expressed on CD45+ immune cells in cancer tissues, and CD4+ T cells and CD8+ T cells were the cell types accounting for most of the total CD73 expression (Fig. 1D). To verify the contributions of these immune cells to the expression of CD73 in tumour tissues, the mRNA expression level of CD73 was detected in sorted CD45- cells, CD45+ cells, CD8+ T cells and CD4+ T cells. However, in both CD45+ cells and T cells, there was no significant difference in the CD73 mRNA level, while CD45- cells from GBM tissues had extremely high CD73 mRNA levels compared to those from LGG, SKCM-brM or NSCLC-brM tissues (Fig. 1E–H). This suggests that the high CD73 expression in T cells may originate from an extracellular source derived from tumour cells. We hypothesised that T cells may take up CD73 in TDEVs originating from GBM. For this reason, we obtained surgical aspiration fluid, CSF and peripheral blood from patients with LGG, GBM, SKCM-brM or NSCLC-brM. The fluids underwent ultrafiltration to obtain EVs, and then, Western blotting and modified ELISA (Fig. 1K) were used to detect CD73 levels in the EVs. As shown in Figs. 1I–L, S1A–C, the EVs extracted from the body fluids of GBM patients had significantly higher CD73 levels than those from the fluids of LGG, SKCM-brM and NSCLC-brM patients. Interestingly, the concentration of CD73 in the EVs from body fluids of GBM patients decreased according to distance from the lesion. This suggests that GBM cells spread to the periphery by releasing CD73 carried by TDEVs, which is taken up by T cells.

### T cells take up CD73+ TDEVs released from GBM cell lines

**Fig. 2:**
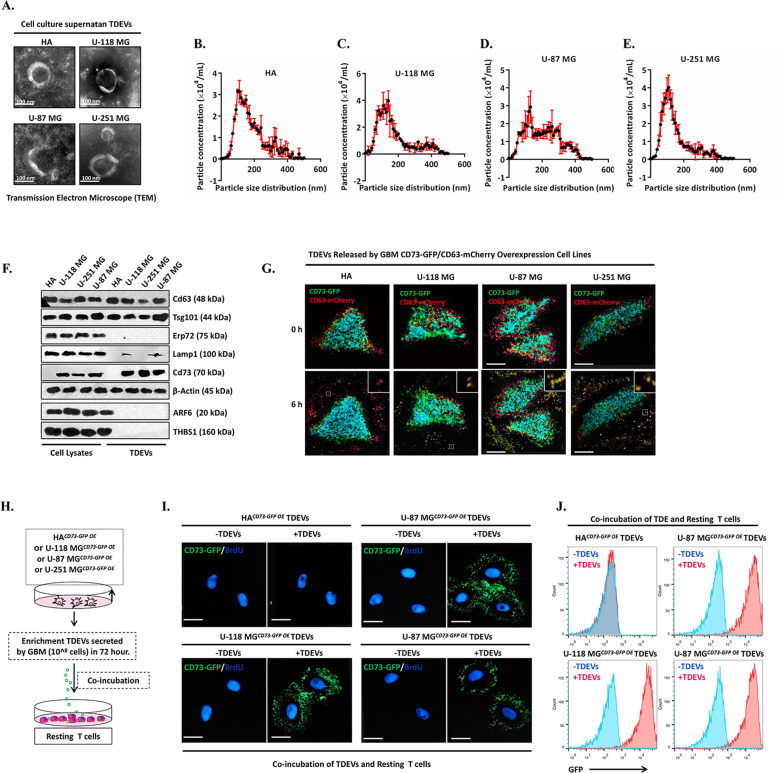
TDEVs released by GBM cells are taken up by T cells. **A** TDEVs in the cell culture supernatant of HAs and U-118 MG, U-87 MG and U-251 MG cells were obtained by ultrafiltration and characterised by transmission electron microscopy. **B**–**E** The count and size of TDEVs in the cell culture supernatant of HAs and U-118 MG, U-87 MG and U-251 MG cells were detected by nanoparticle tracking analysis. **F** Representative immunoblot of the microvesicle marker (ARF6), apoptotic body marker (THBS1), exosome markers (CD63 and TSG101), endoplasmic reticulum negative control marker (Erp72), lysosome negative control marker (Lamp1) and CD73 in cell lysates and TDEVs of HAs and U-118 MG, U-87 MG and U-251 MG cells. **G** Representative confocal microscopy images were used to visualise TDEV release within 6 h from HAs and the three GBM cell lines cotransfected with overexpression plasmids harbouring GFP-tagged CD73 and mCherry-tagged CD63. **H** Schematic diagram of the cocultivation experiment involving HA^*CD73-GFP OE*^, U-118 MG^*CD73-GFP OE*^, U-87 MG^*CD73-GFP OE*^, U-251 MG^*CD73-GFP OE*^ and T cells. **I** Representative confocal microscope images of GFP-labelled nanovesicles taken up by T cells cocultured with HAs and U-118 MG, U-87 MG and U-251 MG cells before and after 12 h. **J** Representative flow cytometry diagram of GFP intensity on the surface of T cells cocultured with HAs, U-118 MG cells, U-87 MG cells. Data are shown as the mean ± SD; *t* test, *p < 0.05 and **p < 0.01 compared to the GBM group. All data are from three independent experiments.

To observe the communication between GBM cells and T cells mediated by CD73+ TDEs, we cultured primary human astrocytes (HAs) and three malignant GBM cell lines (U-118 MG, U-87 MG and U-251 MG). TDEVs in the cell culture supernatant were obtained by ultrafiltration, and characterisation was performed by transmission electron microscopy and nanoparticle tracking analysis (NTA), as shown in Fig. 2A–E. Without any treatment, all HA and GBM cell lines released TDEVs enriched in exosomes, but CD73 was highly expressed in GBM cells and TDEVs compared to HAs (Fig. 2F). Next, overexpression plasmids containing GFP-tagged CD73 and mCherry-tagged CD63 (exosomal marker) were constructed, and HAs and the three GBM cell lines were cotransfected to obtain stably transfected cell lines. Confocal microscopy was used to visualise the release of TDEVs within 6 h. As shown in Fig. 2G, yellow particles, indicating overlap between green and red fluorescence signals on merged images, could be observed around U-118 MG, U-87 MG and U-251 cells, while only red fluorescent particles were observed around HAs, which indicated that GBM cells loaded CD73 protein cargo into EVs (or exosomes) and secreted these vesicles. Next, mCherry-tagged ACTN (EV marker) and mCherry-tagged ARF6 (microvesicle marker) were constructed, and we found that yellow spots indicative of mCherry-ACTN and GFP-CD73 fusion were observed outside of only GBM cells (Supplementary Fig. S2A, B). These data confirm that these yellow fluorescent particles are CD73-positive TDEVs (or exosomes) rather than microvesicles. Next, we cocultured TDEVs from CD73-GFP-overexpressing HAs and GBM cells with T cells, as shown in Fig. 2H, and observed the GFP intensity on T cells to evaluate the uptake of CD73+ TDEVs. Confocal microscopy and flow cytometry showed that the CD73-GFP signal was detected on the surface of T cells after incubation with TDEVs for 12 h (Fig. 2I, J), which indicated that TDEVs secreted from U-118 MG, U-87 MG or U-251 MG cells could be taken up by T cells.

### TDEVs secreted by GBM inhibit T-cell clonal proliferation

**Fig. 3:**
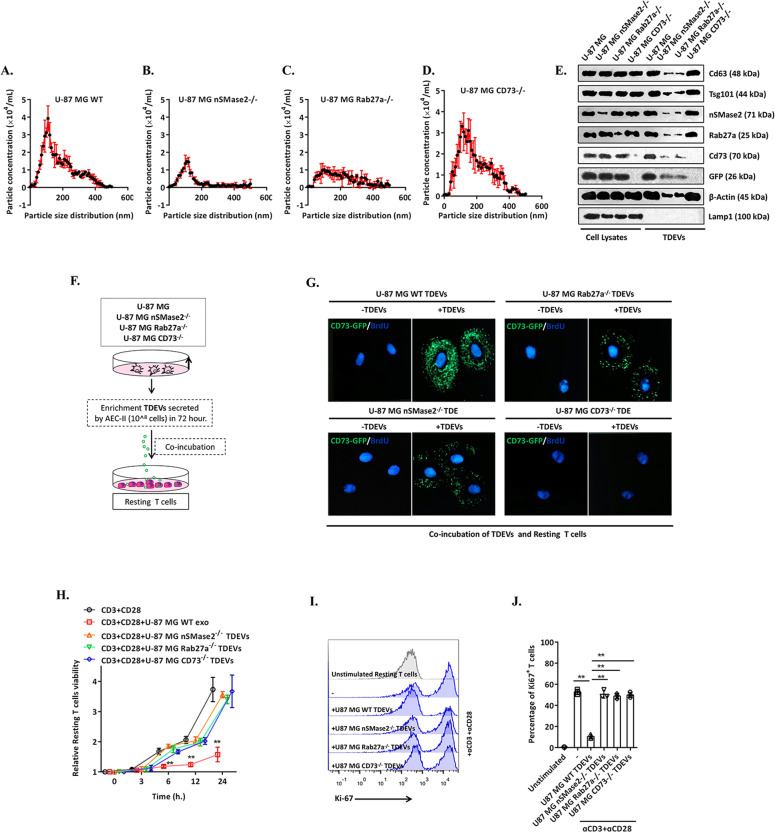
CD73-positive TDEVs released by GBM inhibit T-cell clonal proliferation. **A–D** The count and size of TDEVs in the cell culture supernatant of WT, nSMase2^−/−^, Rab27a^−/−^ and CD73^−/−^ U-87 MG cells were detected by nanoparticle tracking analysis. **E** Representative immunoblot of the expression of CD63, TSG101, lamp1, nSMase2, Rab27a and CD73 in cell lysates and TDEVs of WT, nSMase2^−/−^, Rab27a^−/−^ and CD73^−/−^ U-87 MG cells. **F** Schematic diagram of the cocultivation experiment involving WT, nSMase2^−/−^, Rab27a^−/−^ and CD73^−/−^ U-87 MG GBM cells and T cells. **G** Representative confocal microscopy images of GFP-labelled nanovesicles in T cells cocultured with HAs and U-118 MG, U-87 MG and U-251 MG cells before and after 12 h. **H** WT, nSMase2^−/−^, Rab27a^−/−^ and CD73^−/−^ U-87 MG GBM cell viability was detected by CCK-8 assays in the presence of αCD3 + αCD28. **H** Representative flow cytometry diagram of Ki-67 expression in WT, nSMase2^−/−^, Rab27a^−/−^ and CD73^−/−^ U-87 MG GBM cells in the presence of αCD3 + αCD28. **I** Graph of the percentage of KI-67-positive T cells, related to (**H**). Data are shown as the mean ± SD; *t* test, *p < 0.05 and **p < 0.01 compared to unstimulated T cells. All data are from three independent repeated experiments (**H**–**J**).

The effect of a defect in TDEVs carrying CD73 on T-cell uptake was evaluated. First, two mutant cell lines, nSMase2^−/−^ and Rab27a^−/−^, were constructed on the genetic background of U-87 MG (both proteins are involved in exosome synthesis and secretion). Compared with that from wild-type (WT) U-87 MG cells (Fig. 3A), TDEV release from nSMase2^−/−^ and Rab27a^−/−^ U-87 MG cells was significantly inhibited (Fig. 3B, C). In addition, CD73-deficient U-87 MG cells were constructed, and TDEV release did not differ between these cells and WT U-87 MG cells (Fig. 3D). Western blotting was used to detect cell exosomal markers. As shown in Fig. 3E, the expression of the exosomal markers CD63 and TSG101 in the supernatant of nSMase2^−/−^ and Rab27a^−/−^ U-87 MG cells was significantly downregulated, and although TDEV production was not affected in CD73^−/−^ U-87 MG cells, this deficiency seriously affected the content of CD73 in TDEVs (Fig. 3E). The TDEVs released by the different U-87 MG knockout cell lines were cocultured with T cells in vitro (Fig. 3F). After 12 h, the uptake of CD73-GFP-positive particles by T cells was observed, as shown in Fig. 3G. Compared with the uptake of WT U-87 MG-TDEVs by T cells, that of nSMase2^−/−^ and Rab27a^−/−^ cell-derived TDEVs was affected, and CD73 deficiency markedly altered CD73+ TDEVs uptake by T cells. In addition, we evaluated the uptake of TDEVs secreted by GBM cells expressing mCherry-CD63 (exosomes) and mCherry-ACTN (EVs) (Supplementary Fig. S3A, B).

Next, we observed changes in the physiological effects of T cells after uptake of TDEs. Following stimulation with αCD3 + αCD28 to induce the proliferation of resting T cells, we detected the proliferative activity and expression of the cell cycle entry marker Ki-67, which is expressed in all phases except G0 (Fig. 3 H–J). TDEVs from WT U-87 MG cells almost completely inhibited the T-cell clonal expansion induced by αCD3 + αCD28, while those from U-87 MG cells deficient in nSMase2, Rab27a or CD73 showed reduced inhibitory effects on T-cell clones. These data indicate that CD73+ TDEVs block the clonal proliferation of T cells in vitro.

### CD73+ TDEVs interfere with T-cell aerobic glycolysis and clonal proliferation

**Fig. 4:**
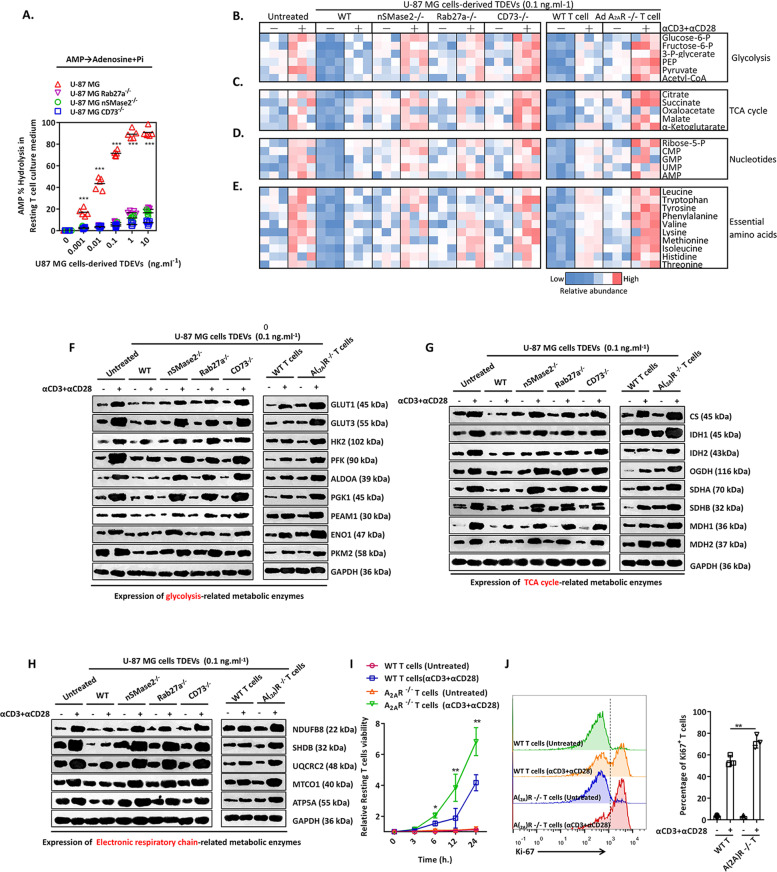
CD73-positive TDEVs inhibit T-cell aerobic glycolysis in an adenosine–A_2A_R-dependent mechanism. **A** Adenosine level was detected by ELISA in culture medium from T cells cocultured with TDEs from WT, nSMase2^−/−^, Rab27a^−/−^ and CD73^−/−^ U-87 MG GBM cells (*n* = 5). **B**–**E** The relative abundance of polar metabolites from glycolysis and TCA cycle, nucleotides and essential amino acids was detected by liquid phase-mass spectrometry in T-cell lysates exposed to TDEs from WT, nSMase2^−/−^, Rab27a^−/−^ and MG CD73^−/−^ U-87 GBM cells in the presence or absence of αCD3 + αCD28 (*n* = 3). **F**–**H** Representative immunoblot of the expression of metabolic enzymes related to glycolysis, the TCA cycle and electron transport chain as detected by western blotting in T-cell lysates supplemented with TDEs from WT, nSMase2^−/−^, Rab27a^−/−^ and CD73^−/−^ U-87 MG GBM cells in the presence or absence of αCD3 + αCD28. **I** The viability of WT and A_2A_R^−/−^ T cells was detected in the presence or absence of αCD3 + αCD28. **J** Ki-67 expression was detected in WT and A_2A_R^−/−^ T cells in the presence or absence of αCD3 + αCD28. Data are shown as the mean ± SD; *t* test, *p < 0.05 and **p < 0.01 compared to unstimulated T cells. All data are from three independent experiments (**F**–**J**).

The CD39/CD73 interaction has been confirmed to calibrate the duration of immune cell purine signalling by catalysing the conversion of ATP/ADP/AMP/Pi + adenosine [13]. Adenosine can inhibit T-cell immune function [5] and metabolic proliferation [14]. To this end, we next investigated whether CD73+ TDEVs from U-87 MG cells can interfere with the energy metabolism of T cells through adenosine. In T-cell culture medium supplemented with U-87 MG-TDEs, the level of adenosine was detected to evaluate the AMP hydrolysis efficiency of CD73+ TDEs. As shown in Fig. 4A, WT U-87 MG-TDEVs rapidly hydrolysed AMP to produce excess adenosine, while TDEVs derived from nSMase2^−/−^, Rab27a^−/−^ or CD73^−/−^ U-87 MG cells did not substantially accelerate the conversion of AMP to adenosine and Pi in T-cell culture medium. To undergo DNA replication and mitosis after entering G1 phase, cells must have adequate nutrients to pass metabolic checkpoints [15]. Aerobic glycolysis is the main source of synthetic metabolites in T cells stimulated by αCD3 + αCD28 [16]. Cell lysates were analysed for polar metabolites by a liquid phase-mass spectrometer. As shown in Fig. 4B, in αCD3 + αCD28-stimulated T-cell clones, the accumulation of glycolytic metabolites increased 2.87-fold compared with that in resting T cells. A similar increase (3.36-fold) was observed for the tricarboxylic acid (TCA) cycle (Fig. 4C). In addition, pentose phosphate pathway metabolites were significantly upregulated (4.33-fold) (Fig. 4D), which are critical for the synthesis of essential amino acids (3.62-fold increase) (Fig. 4E) and nucleotides during T-cell proliferation. Pretreatment of resting T cells with WT U-87 MG-TDEVs significantly reduced the abundance of polar metabolites in αCD3 + αCD28-induced aerobic glycolysis and the pentose phosphate pathway, while U-87 cells deficient in nSMase2, Rab27a or CD73 showed reduced inhibition of T-cell metabolism by TDEVs (Fig. 4B–E). In addition, we constructed adenosine receptor 2 A (A_2A_R, an adenosine receptor expressed on T cells)-deficient T cells and evaluated whether CD73 + TDEVs inhibit T-cell metabolism in a manner that requires adenosine-A_2A_R. As shown in Fig. 4B–E, A_2A_R-deficient T cells were resistant to WT U-87 MG-TDEs, which inhibited the accumulation of polar metabolites of aerobic glycolysis and the pentose phosphate pathway. Next, we detected the expression of related enzymes involved in the regulation of aerobic glycolysis. Glut1 and Glut3 control glucose uptake in cells, and among others, hexokinase 2 (HK2) and phosphofructokinase (PFK) control the reaction speed of aerobic glycolysis. These glycolysis-related enzymes, which were significantly upregulated by stimulation with αCD3 + αCD28, were inhibited after pretreatment with WT U-87 MG-TDEVs, while TDEVs from U-87 MG cells deficient in nSMase2, Rab27a or CD73 had no effect on the expression of glycolysis-related enzymes. Similar phenomena were observed for the expression of enzymes related to the TCA cycle and the electron transport chain. As shown in Fig. 4G, H, CD73 + TDEVs from U-87 MG cells significantly interfered with mitochondrial oxygen metabolism and oxidative phosphorylation in T cells. In A_2A_R-deficient T cells, U-87 MG-TDEVs had no significant effect on the expression of enzymes related to glycolysis, the TCA cycle or the electron transport chain (Fig. 4F–H). Furthermore, these deficiencies rescued the inhibition of T-cell clonal proliferation by TDEVs derived from U-87 MG cells (Fig. 4I, J). These data indicate that TDEVs from GBM cells fuse with the surface of T cells, increase the concentration of adenosine around T cells, activate A_2A_R and inhibit aerobic glycolysis in T cells, thereby inhibiting the production of energy-carrying substances needed for T-cell clonal proliferation.

### Elimination of CD73+ TDEVs reduces tumour proliferation in GBM tumour-bearing mice

**Fig. 5:**
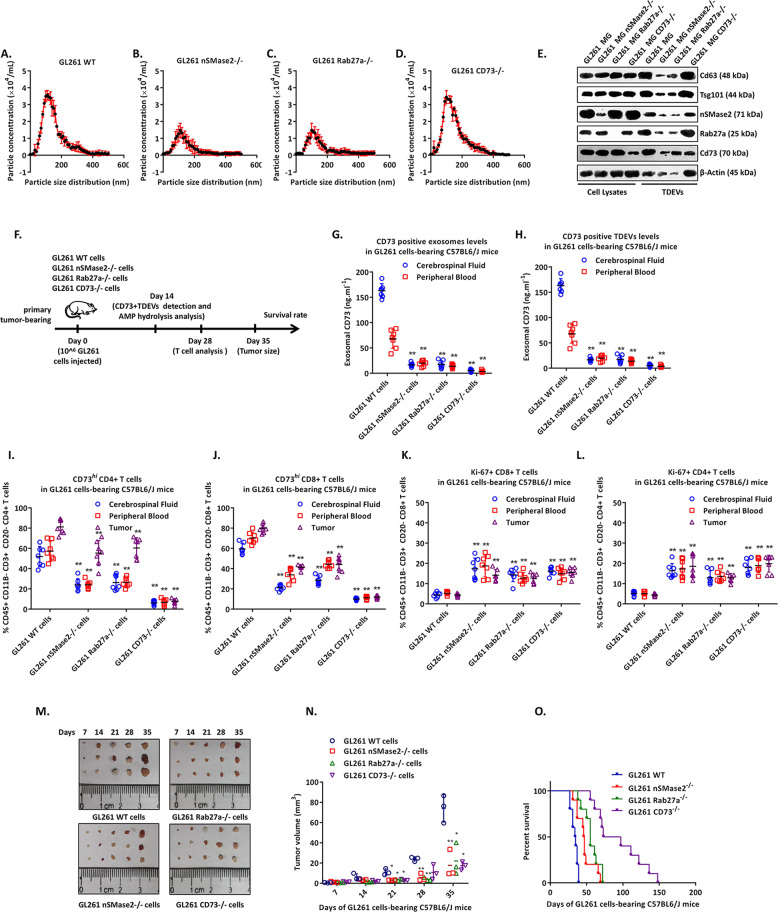
The elimination of CD73-positive TDEVs inhibits the proliferation of GBM cells in vivo. **A**–**D** The count and size of TDEVs in the culture supernatant of WT, nSMase2^−/−^, Rab27a^−/−^ and CD73^−/−^ GL-261 cells were detected by nanoparticle tracking analysis. **E** Representative immunoblot of the expression of exosome markers (CD63 and TSG101), nSMase2, Rab27a and CD73 in cell lysates and TDEVs of WT, nSMase2^−/−^, Rab27a^−/−^ and CD73^−/−^ GL-261 cells. **F** Experimental protocol for mice bearing WT, nSMase2^−/−^, Rab27a^−/−^ or CD73^−/−^ GL-261 cell-derived tumours. **G** The levels of CD73-positive TDEVs in the cerebrospinal fluid and peripheral blood of WT, nSMase2^−/−^, Rab27a^−/−^ and CD73^−/−^ GL-261 tumour-bearing mice (*n* = 6). **H** Adenosine levels in the cerebrospinal fluid and peripheral blood of WT, nSMase2^−/−^, Rab27a^−/−^ and CD73^−/−^ GL-261 tumour-bearing mice (*n* = 6). **I**–**L** The percentage of CD73^*hi*^CD4^+^, CD73^*hi*^CD8^+^, Ki67^+^CD4^+^ and Ki67^+^CD8^+^ T cells in the cerebrospinal fluid, peripheral blood and tumours of WT, nSMase2^−/−^, Rab27a^−/−^ and CD73^−/−^ GL-261 tumour-bearing mice (*n* = 6). **M**, **N** The tumour volume in mice bearing WT, nSMase2^−/−^, Rab27a^−/−^ or CD73^−/−^ GL-261 cell-derived tumours at 7, 14, 21, 28 and 35 days after implantation (M) (*n* = 3). **O** Survival curve of tumour-bearing mice inoculated with WT, nSMase2^−/−^, Rab27a^−/−^ or CD73^−/−^ GL-261 cells (*n* = 10). Data are shown as the mean ± SD; *t* test, *p < 0.05 and **p < 0.01.

Next, we verified whether eliminating CD73+ TDEVs has an inhibitory effect on orthotopic tumours in vivo. The GL-261 mouse GBM cell line was used to construct a tumour-bearing mouse model. In vitro, GL-261 cells in which nSMase2, Rab27a or CD73 was knocked out released levels of TDEVs consistent with those released by human GBM cells (Fig. 5A–E). WT, nSMase2^−/−^, Rab27a^−/−^ or CD73^−/−^ GL-261 cells were implanted into the brain of C57BL/6J mice to observe differences in tumour growth (Fig. 5F). After 14 days, CSF and blood were collected from GL-261 tumour-bearing mice to detect the levels of CD73+ TDEVs and adenosine. As shown in Fig. 5G, H, lower concentrations of CD73+ TDEVs and adenosine were detected in the CSF and blood of nSMase2^−/−^, Rab27a^−/−^ and CD73^−/−^ GL-261 tumour-bearing mice than in those of WT GL-261 tumour-bearing mice. After 28 days, tumour tissue, CSF and blood were collected from GL-261 tumour-bearing mice to detect T cells; as shown in Fig. 5I–L, lower proportions of CD73^*hi*^Ki67^+^CD4^+^ or CD73^*hi*^Ki67^+^CD8^+^ T cells were detected in tumour tissue, CSF and blood from nSMase2^−/−^, Rab27a^−/−^ and CD73^−/−^ GL-261 tumour-bearing mice than from WT GL-261 tumour-bearing mice. Tumours were harvested at different times, tumour size was recorded, and the survival period of the remaining mice not sampled during surgery was determined (Fig. 5M–O). In WT GL-261 tumour-bearing mice, tumours exceeding 0.2 cm^2^ were detected after 3 weeks, and the longest survival time was 39 days. In contrast, among nSMase2^−/−^, Rab27a^−/−^ and CD73^−/−^ GL-261 tumour-bearing mice, only tumours smaller than 0.2 cm^2^ were collected after 3 weeks, tumours appeared to stop growing in the following 2 weeks, and the longest survival times were 69, 72 and 148 days, respectively. These data indicate that targeting CD73+ TDEVs can reduce the tumour growth rate and improve the central and peripheral immunosuppressive microenvironments in GBM tumour-bearing mice.

## Discussion

In recent years, a variety of immunotherapies have been used in the treatment of different solid tumours. New research ideas have shifted from directly killing cancer cells to improving the activity of a patient’s own immune system. For example, ICT, which aims to target molecules highly expressed in tumours, such as PD-L1/PD-1 and CTLA-4, to reduce the depletion of effector T cells [17], is especially effective in SKCM [18] and NSCLC [19]. However, in highly invasive GBM, a single ICT often fails to achieve good results in regard to the growth of GBM tumours [20], which is often attributed to the fact that GBM is a highly immunosuppressive malignant tumour. Immunosuppression in GBM occurs throughout the body, especially in the brain and peripheral immune organs. Traditional chemotherapy aggravates the suppression of the systemic immune system [1]. In our previous study, the level of Ki-67 + CD8+ T cells was significantly reduced in the CSF of GBM patients, while LGG patients had a higher percentage of Ki-67 + CD8+ T cells in the CSF than healthy subjects [7]. This indicated that GBM has other mechanisms, such as directly inhibiting the clonal proliferation of T cells, to offset the therapeutic effect of ICT. Recently, CD73 has attracted much attention as an auxiliary ICT target. CD73-deficient mice have achieved benefits with ICT [10]. Anti-CD73 antibodies have also achieved remarkable results in preclinical and early clinical studies [21, 22].

In this study, we detected high levels of CD73+ TDEVs in the lesions, CSF and blood of GBM patients compared to those of LGG and metastatic brain cancer patients. High CD73 expression levels can be detected on the surface of CD45+ immune cells, especially CD4+ and CD8+ T-lymphocytes (fold change compared to tumour-resident macrophages (TRMs), DCs, and neutrophiles: 46, 26 and 567 and 15, 8.4 and 181, respectively). However, it remains unknown whether the origin of CD73 on the surface of these T-lymphocytes is external or internal. In fact, as early as 2011, studies found that CD73+ TDEVs secreted by a variety of cancer cells inhibit the immune activity of T cells in vitro [5]; in addition, high levels of CD73 expression can be detected in GBM and are related to the poor prognosis of GBM [4, 23, 24]. Our research showed that these CD73+ TDEVs enriched in exosomes originate from GBM cells and are taken up by T-lymphocytes. Importantly, coculture of CD73+ TDEVs with T cells attenuated the T-cell clonal proliferation induced by αCD3 + αCD28. It is well known that T-lymphocytes undergo clonal proliferation when antitumour immunity is activated. After preparing a variety of nutrients in the G0 phase, cells enter the cell division cycle. Before that, T-lymphocytes mainly utilise aerobic glycolysis to obtain energy and necessary metabolites. In this study, we observed that CD73+ TDEVs directly interfered with the energy metabolism of T cells in vitro, reducing the levels of the glucose transfer receptors Glut1 and Glut3 and aerobic glycolysis and inhibiting the expression of related enzymes in aerobic glycolysis, the TCA cycle and the electron transport chain.

The activity of CD73 is related to the hydrolysis of 5′-AMP- to produce adenosine, an important mediator that regulates cell activity in various diseases, such as sepsis [25], asthma [26] and ischaemia-reperfusion injury [27, 28]. In the tumour microenvironment, adenosine also inhibits the immune response and supports immune escape by cancer cells [29]. In vitro, CD73+ TDEVs can effectively reduce the level of surrounding AMP in the T-cell environment and increase the concentration of adenosine, which has been found in the CSF of GBM patients and tumour-bearing mice. Adenosine receptors (four subtypes: A_1_, A_2A_, A_2B_ and A_3_) are widely expressed in immune cells, and they can precisely regulate the immune response of cells through affinity transformation [30]. In T cells, the high-affinity A_2A_ receptor is mainly expressed [31]. After migration to the tumour environment, A_2A_R activation triggers the production of cAMP and the dephosphorylation of STAT5, which abolishes IL-2 and TCR signal-mediated T cell-mediated immune effects [32]. In addition, disruption of mTORc1 activation in T cells is related to the adenosine-A_2A_R interaction [33], which is essential for the clonal proliferation of T cells [34]. After we knocked out A_2A_R in T cells, CD73+ TDEVs lost the capacity to inhibit T-cell proliferation, and T cells resumed aerobic glycolysis stimulated by αCD3 + αCD28.

CD73/CD39 catalyses a two-step process to produce adenosine. CD39 converts extracellular ATP (or ADP) into 5′-AMP, and CD73 subsequently converts 5′-AMP into adenosine. Mouse Treg cells express CD39 and CD73, and their synergistic enzymatic action produces adenosine in an immunosuppressive mechanism independent of IL-10 and TGF-β [35, 36]. In this study, we confirmed that CD73 carried by EVs derived from central GBM has a systemic immunosuppressive effect on T cells. We found that CD73+ TDEVs not only fill the GBM centre but also are widely distributed in the periphery. For cancer cells, suppressing immune cells via secretion of exosomes carrying CD73 has a relatively efficient immune escape-promoting effect. This is because exosomes not only have an extremely small particle size and fat solubility but can also effectively penetrate the blood-brain barrier and affect the whole body. Importantly, exosomes can carry a variety of immunosuppressive molecules to coordinate and target systemic immune cells. An important study showed that GBM inhibited the activity of T cells in mouse lymphatic vessels by releasing exosomes containing PD-L1 on the surface, which induced the suppression of systemic antitumour immunity and immune memory [37]. Our research also confirmed that LGALS9+ EVs released by GBM can inhibit antigen processing and presentation in peripheral DCs [7]. We blocked the secretion of TDEVs and the expression of CD73 in GBM cells, which alleviated tumour proliferation in tumour-bearing mice and prolonged survival. In conclusion, we found that CD73-positive TDEVs enriched in exosomes derived from GBM cells inhibit T-cell aerobic glycolysis, cell cycle entry and clonal proliferation systemically through an adenosine-A_2A_R interaction-dependent process, which indicates that treatments addressing the activity of CD73+ TDEVs have the potential to enhance ICT.

## Supplementary information

Supplementary Figure Legends

Supplementary Figure 1

Supplementary Figure 2

Supplementary Figure 3

Supplementary Table1. Clinical characteristics of brain tumour samples

Supplementary Table2. Information of antibody

Supplementary Table3. sgRNA oligonucleotides

## Data Availability

The authors declare that the data supporting the findings of this study are available within the paper and its Supplementary Information Files. Additional data related to this study may be requested from the authors.
