# Supplementary material for: CD73-positive extracellular vesicles promote glioblastoma immunosuppression by inhibiting T-cell clonal expansion
Source: Cell Death Dis. 2021 Nov 9;12(11):1065. doi: 10.1038/s41419-021-04359-3 (PMC8578373; doi:10.1038/s41419-021-04359-3)
Supplement: Supplementary file 1 — Supplementary Figure Legends [file 41419_2021_4359_MOESM1_ESM.docx]

**Supplementary Figure Legends**

**Supplementary Figure 1. Quantification of CD73 in EVs from patients with brain tumours.**

(a) CD73 levels in EVs of body fluids from brain tumour patients; CD63 was used as the exosome internal control, ACTN was used as the Pan-EV control, and the BCA method was used to determine the EV loading volume (n_GBM_=17, n_LGG_=13, n_SKCM-BrM_=5, and n_NSCLC-BrM_=4).

(b and c) The concentration of CD63 and P-selectin-positive EVs in cerebrospinal fluid, peripheral blood and surgical aspiration fluid from brain tumour patients (n_GBM_=17, n_LGG_=13, n_SKCM-BrM_=5, and n_NSCLC-BrM_=4).

Data are shown as the mean ± SD; t-test, *p < 0.05 and ** p < 0.01 compared to the GBM group in panels b and c.

**Supplementary Figure 2. CD73 in microparticles and EVs released by GBM cells.**

(a and b) Representative confocal microscopy images to visualize TDEV release within 6 h from HAs and three GBM cell lines cotransfected with overexpression plasmids encoding GFP-tagged CD73 and mCherry-tagged ACTN or ARF6. All data are from 3 independent experiments.

**Supplementary Figure 3. TDEV uptake by T cells.**

(a and b) Representative confocal microscopy images of the uptake of mCherry-CD63^+^ exosomes and mCherry-ACTN^+^ EVs by T cells cocultured with HAs and U-118 MG, U-87 MG and U-251 MG cells before and after 12 h. All data are from 3 independent experiments.
