# Supplementary figures and images for: CD73-positive extracellular vesicles promote glioblastoma immunosuppression by inhibiting T-cell clonal expansion
Source: Cell Death Dis. 2021 Nov 9;12(11):1065. doi: 10.1038/s41419-021-04359-3 (PMC8578373; doi:10.1038/s41419-021-04359-3)

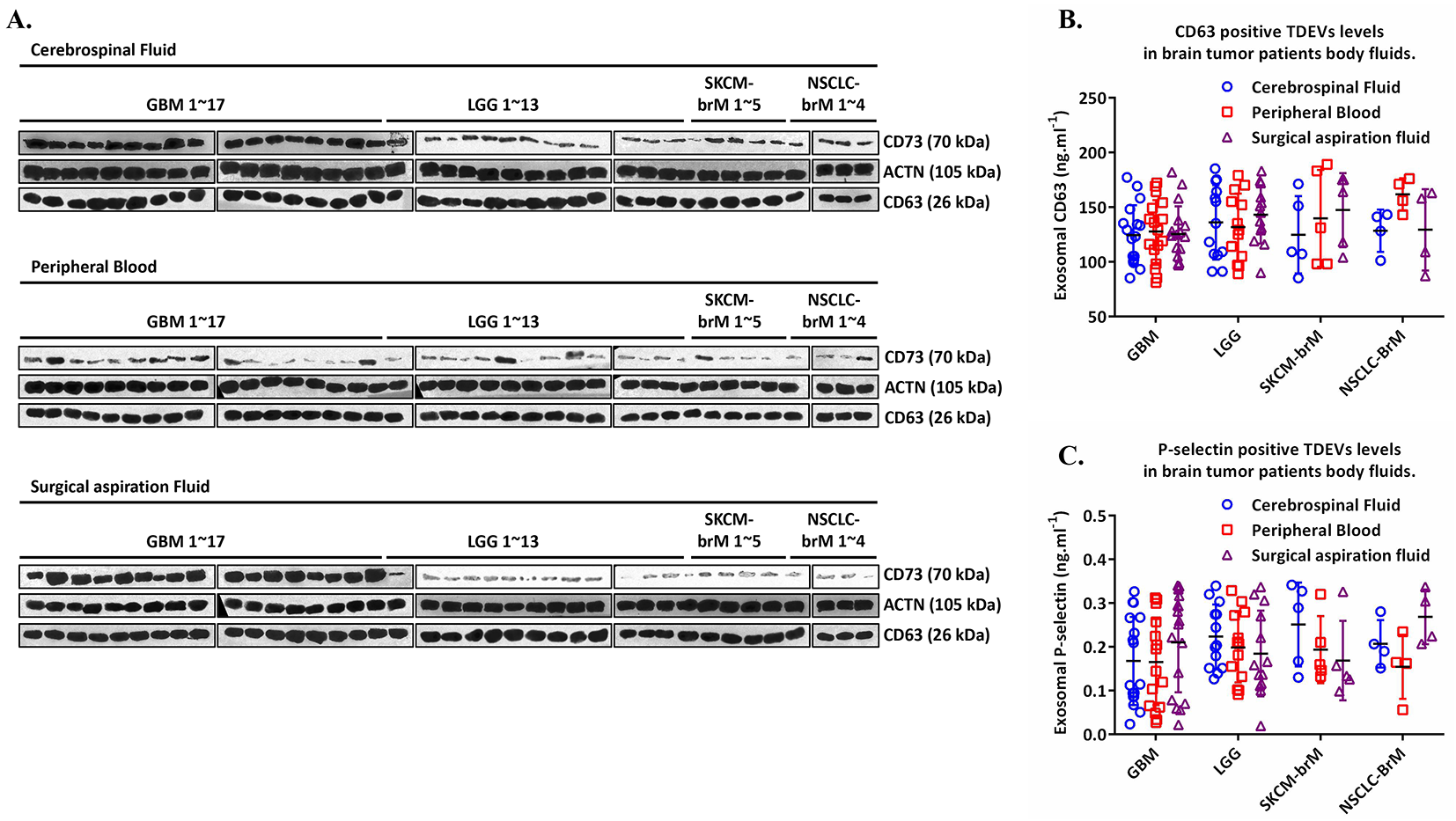

Supplement: Supplementary file 2 — Supplementary Figure 1 [file 41419_2021_4359_MOESM2_ESM.tif]

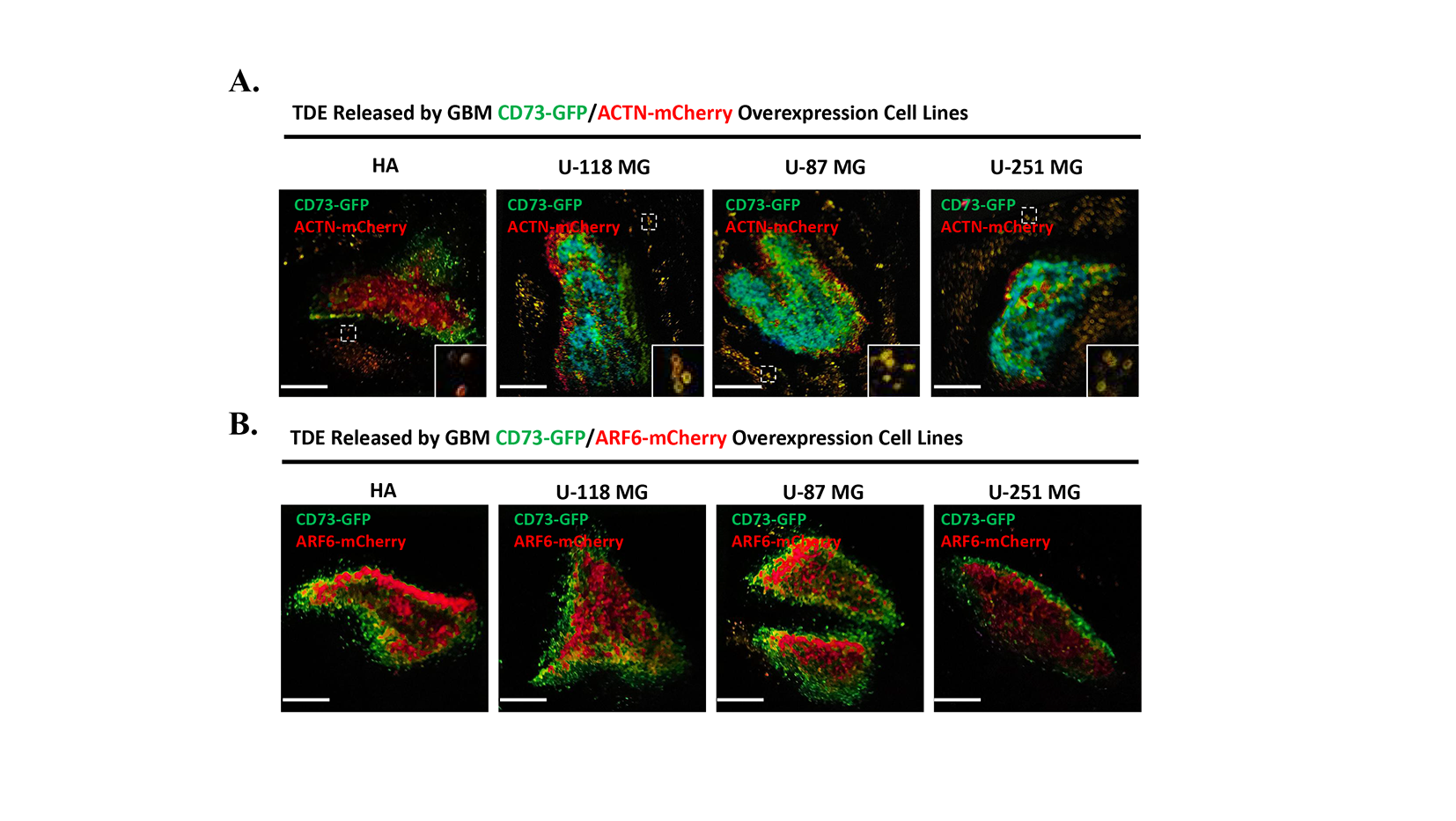

Supplement: Supplementary file 3 — Supplementary Figure 2 [file 41419_2021_4359_MOESM3_ESM.tif]

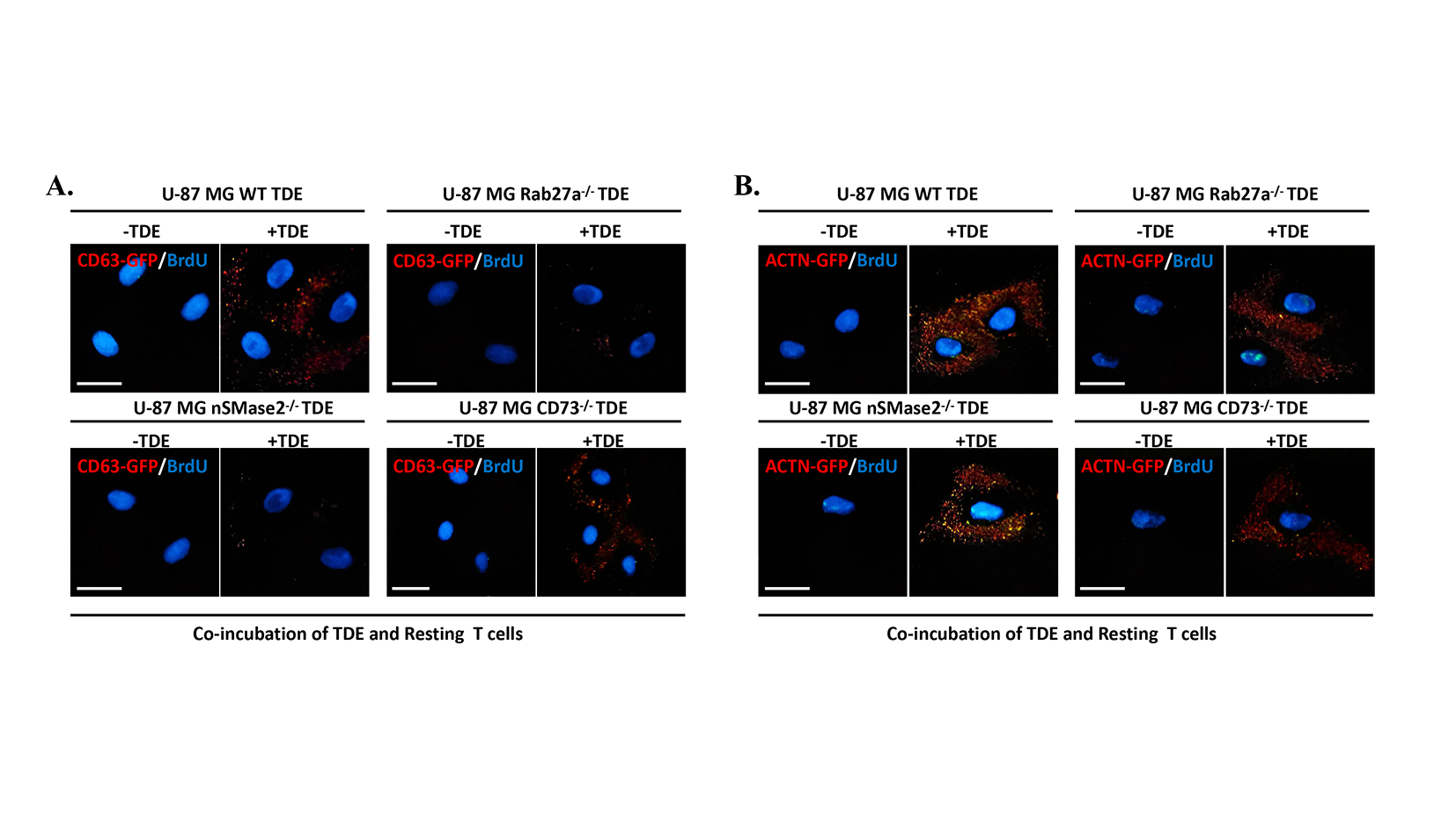

Supplement: Supplementary file 4 — Supplementary Figure 3 [file 41419_2021_4359_MOESM4_ESM.tif]
