# Supplementary material for: CD73-positive extracellular vesicles promote glioblastoma immunosuppression by inhibiting T-cell clonal expansion
Source: Cell Death Dis. 2021 Nov 9;12(11):1065. doi: 10.1038/s41419-021-04359-3 (PMC8578373; doi:10.1038/s41419-021-04359-3)
Supplement: Supplementary file 7 — Supplementary Table3. sgRNA oligonucleotides [file 41419_2021_4359_MOESM7_ESM.docx]

| **sgRNA oligonucleotides:** | **Sequences:** |
| --- | --- |
| human NT5E guide 1: | 3’-CCGCTTTAGAGAATGCAACA -5’ |
| human NT5E guide 2: | 3’-AGGCAATCCACCTTCCAAAG -5’ |
| human Rab27a guide 1: | 3’-CCAAAGCTAAAAACTTGATG-5’ |
| human RAB27a guide 2: | 3’-CAACAGTGGGCATTGATTTC-5’ |
| human nSMase2 guide 1: | 3’-GAGAAACGCAAAGGGCAGCG-5’ |
| human nSMase2 guide 2: | 3’-CGGTCCACCAGCCAGTAGCA-5’ |
| mouse NT5E guide 1: | 3’-GCGCAAACATTAAGGCACGG-5’ |
| mouse NT5E guide 2: | 3’-TGAATAAGATCATCGCCCTG -5’ |
| mouse RAB27a guide 1: | 3’-CCAAGGCCAAGAACTTGATG-5’ |
| mouse RAB27a guide 2: | 3’-CACAGTGGGCATTGATTTCA-5’ |
| mouse nSMase2 guide 1: | 3’-CGTTAATGGCCGACTGGCTC-5’ |
| mouse nSMase2 guide 2: | 3’-AATGCCAAGTGGTTAAAGGA-5’ |
| human A2aR guide 1 | 3’-ACTCGATCTTCAGATAGCCT-5’ |
| human A2aR guide 2 | 3’-TACCATGGCATCGTAGCGCA-5’ |
